# Variable Direct Electromechanical Properties of As-Electrospun Polystyrene Microfiber Mats with Different Electrospinning Conditions

**DOI:** 10.3390/polym14091840

**Published:** 2022-04-29

**Authors:** Chonthicha Iumsrivun, Kazuki Matsuda, Shunsaku Ohkubo, Yuya Ishii

**Affiliations:** Faculty of Fiber Science and Engineering, Kyoto Institute of Technology, Kyoto 606-8585, Japan; d9851501@edu.kit.ac.jp (C.I.); m0651020@edu.kit.ac.jp (K.M.); m0651004@edu.kit.ac.jp (S.O.)

**Keywords:** microfiber, electrospinning, electromechanics, deposition time, electret

## Abstract

As-electrospun microfiber mats comprising atactic polystyrene (aPS), a low-cost commodity polymer, have demonstrated beneficial electromechanical properties. However, the variability of the electromechanical properties of fiber mats produced using different electrospinning conditions has not been investigated. Therefore, herein, the direct electromechanical properties of aPS fiber mats produced using different deposition times (*t*_dep_) and electrospinning voltages (*V*_ES_) are investigated. The resulting apparent piezoelectric *d* constant (*d*_app_) of the fiber mats demonstrates a specific peak value for *t*_dep_ as high as ~1600 pC N^−1^ under 1-kPa pressure application after ~0.2-kPa pre-pressure application, although the *d*_app_ of the fiber mats produced with some conditions is nearly zero pC·N^−1^. Furthermore, the peak position of *d*_app_ with *t*_dep_ is fundamentally determined with *σ*_Eff0_/*Y*_D_(*h*-*h*_pre_) [*σ*_Eff0_: effective surface charge density, *Y*_D_(*h*-*h*_pre_): secant modulus of elasticity]. Charge distribution models for fiber mats with different *t*_dep_ are established. The models explain the characteristics of the significant changes in *Y*_D_(*h*-*h*_pre_) and *σ*_Eff0_ with *t*_dep_. These findings provide significant directions for the production of fiber mats with improved direct electromechanical properties.

## 1. Introduction

Recently, cyber physical systems (CPSs) have attracted significant attention because they collect a large amount of information from physical spaces, analyze this information in cyberspace, and provide useful feedback to people and society. These systems employ a huge number of sensors, which are essential for connecting the physical and cyber spaces. Among them, soft pressure sensors are a key type of sensor, and are well-suited to monitoring the pressure signals (movement, vitals, touching, and grabbing/releasing) of humans, animals, and soft robots [1,2,3,4,5,6,7].

Nano/microfiber mats are considered promising materials for the development of soft pressure sensors owing to their ultra-light weight, flexibility, and breathability. Many types of soft pressure sensors based on electrospun nano/microfiber mats have been reported, such as piezoresistive [5], piezocapacitive [8,9], triboelectric [10,11,12], piezoelectric [13,14,15], and piezoelectret sensors [16,17]. The applications of triboelectric, piezoelectric, and piezoelectret sensors are expected to drastically increase, considering that they enable self-power-generative pressure sensing, which resolves the problem of covering the operational energies of the sensors [18,19,20]. Although triboelectric sensors provide a high-power output with several material combinations [20], they require a triboelectrification procedure in direct contact with triboelectrification layers, which may result in an unstable output for pressure sensing. Piezoelectric sensors have been studied extensively [18], considering that they output steady signals for each pressure application. Piezoelectric materials use expensive piezoelectric polymers, such as poly(vinylidene fluoride) (PVDF) [5,21,22], poly(vinylidenefluoride-co-trifluoroethylene) (PVDF-TrFE) [15], and poly(L-lactic acid) (PLLA) [23,24], and require post-treatment, such as polling, heating, and heat-drawing in order to improve their piezoelectric properties.

Iumsrivun et al. [25] reported that as-electrospun fiber mats comprising several piezoelectric/nonpiezoelectric polymers—including poly(d,l-lactic acid), poly(methyl methacrylate), poly(l-lactic acid), poly((*R*)-3-hydroxybutyric acid), and atactic polystyrene (aPS)—demonstrated direct electromechanical properties. They found that over time, the as-electrospun aPS fiber mat demonstrated significantly high direct electromechanical responses with an apparent piezoelectric *d* constant (*d*_app_) of ≤3350 pC·N^−1^ and stable electromechanical characteristics. Furthermore, Ishii et al. [26] demonstrated the excellent converse electromechanical responses of the aPS fiber mat with a *d*_app_ of >30,000 pm·V^−1^. Therefore, the as-electrospun aPS fiber mat is considered to be a promising high-performance platform for pressure sensors or actuators, considering that aPS is a low-cost commodity polymer and the aPS fiber mat does not require post-treatments. These fiber mats can be categorized as being of the piezoelectret type, considering that piezoelectric polymers are not used as materials, and the origin of the operation is reported as a real charge [25]. However, despite these investigations, no study has yet reported the electromechanical properties of the aPS fiber mats produced under different electrospinning conditions.

Electrospinning exhibits several fabrication parameters, such as the polymer solution concentration, solvent type, spinning voltage, deposition time, solution feed rate, and the distance between the needle and the collector. All of these parameters are known to affect the geometrical characteristics of electrospun fibers [13,22,27]. However, no study has yet reported the effect of the deposition time (*t*_dep_) and spinning voltage (*V*_ES_) on the electromechanical properties of electrospun fiber mats. Therefore, in this study, as-electrospun aPS fiber mats are fabricated with different *t*_dep_ and *V*_ES_ values in order to investigate their direct electromechanical properties; the property demonstrates a specific peak value for the different fabrication conditions. Additionally, the origins of the different resulting direct electromechanical properties are investigated. The findings of this study provide guidance for the production of fiber mats with improved direct electromechanical properties, and pave the way for the development of high-performance pressure sensors that are flexible, lightweight, and support low-cost/large-area productions.

## 2. Materials and Methods

### 2.1. Materials and Fabrication of the Fiber Mats

**Figure 1:**
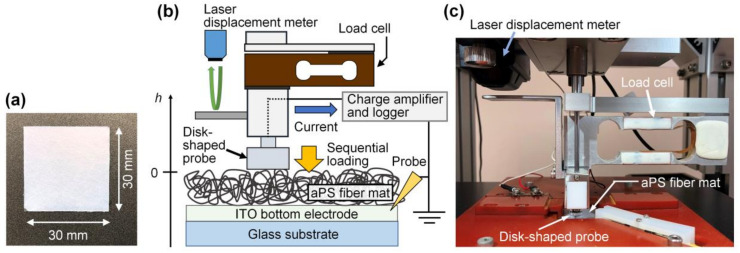
(**a**) Photograph of the entire as-electrospun aPS fiber mat on the ITO/glass substrate. (**b**) A schematic of the sequential approaching/loading technique using the piezoelectric constant measurement apparatus. (**c**) A photograph of the apparatus.

Atactic polystyrene (aPS; *M*w ≈ 280,000) pellets and *N*,*N*-dimethylformamide (DMF) were purchased from Merck KGaA and Nacalai Tesque, Inc., respectively. The aPS pellets were dissolved in DMF at room temperature for approximately 24 h in order to prepare a 30 wt% aPS solution. Then, the aPS solution was loaded into a syringe equipped with a stainless-steel needle (inner diameter = 0.34 mm). The solution was electrospun with different *t*_dep_ and *V*_ES_, as summarized in Table 1, at room temperature. The electrospun fibers were directly deposited on a poly(ethylene terephthalate) (PET) substrate coated with a ~0.10-μm-thick indium tin oxide (ITO) layer (749753-5EA, Merck KGaA, Darmstadt, Germany) for cross-sectional field-emission scanning electron microscopy (FESEM), and a glass substrate coated with a ~0.15-μm-thick ITO (1006, GEOMATEC Co., Ltd., Yokohama, Japan) layer for other measurements. The dimensions of the ITO/glass and ITO/PET substrates (length × width × thickness) were 30 mm × 30 mm × 0.7 mm and 30 mm × 30 mm × 0.18 mm, respectively (Figure 1a). The distance between the tip of the needle and the ITO surface was 20 cm for all of the electrospinning experiments. Each feed rate was optimized in order to ensure stable electrospinning.

### 2.2. Morphological and Electrical Characterization

Optical microscopy and FESEM images were obtained using an optical microscope (IX71, Olympus Corp., Tokyo, Japan) and a field-emission scanning electron microscope (JSM-7001F, JEOL Ltd., Tokyo, Japan), respectively. A ~2-nm-thick layer of gold was coated onto each fiber before the FESEM observation of the individual fibers, whereas ~5- and ~2-nm-thick layers of gold were coated on the top and cross-section of each fiber mat, respectively, before the cross-sectional FESEM observation. The average diameter of the fiber was determined based on the FESEM observation results of 100 fibers. The average thickness of each fiber mat ($T_{F0})$ was determined from the 50-thickness measurements from the cross-sectional optical microscopy images of each fiber mat.

### 2.3. Quasistatic Direct Electromechanical Characterization

The quasistatic direct electromechanical properties of each aPS fiber mat were measured at 7 days after fabrication using the sequential approaching/loading technique, with a piezoelectric constant measurement apparatus (PF-02B, Lead Techno Co., Ltd., Otsu, Japan) equipped with a laser confocal displacement meter (LT-9010M/LT-9500, Keyence Corp., Osaka, Japan), as shown in Figure 1b,c. Further details of this technique were presented in a previous study [28]. Briefly, a disk-shaped metallic probe with a diameter of 8.0 mm was placed on the surface of each aPS fiber mat and indented perpendicular to the surface. Here, the probe acted as a top electrode. The displacement of the probe (*h*), the load applied [*F*(*h*)], and the amount of charge flowing from the metallic probe to the bottom ITO electrode [*Q*(*h*)] (Figure 1 shows the positive directions of *h* and the current) were simultaneously measured for approximately 20 s using a laser confocal displacement meter, a load cell, and a charge amplifier, respectively. The pressure applied to the fiber mat [*P*(*h*)] was calculated as *P*(*h*) = *F*(*h*)/*S*, where *S* is the area of the probe (~5.0 × 10^−5^ m^2^). *h* = 0 was determined as the height at which the metallic disk probe came into contact with the fiber mat.

### 2.4. Electrostatic Characterization

The surface potential of each fiber mat was measured at 7 days after fabrication using a digital low-voltage static meter (KSD-3000, Kasuga Denki, Inc., Kawasaki, Japan) with a 20 × 20 mm measurement area. Each bottom ITO electrode was electrically grounded. Furthermore, the total amount of stored charge in each fiber mat was measured with a coulomb meter (NK-1001A, Kasuga Denki, Inc., Kawasaki, Japan. and KQ-1400, Kasuga Denki, Inc., Kawasaki, Japan).

## 3. Results and Discussion

### 3.1. Geometrical Characteristics

**Figure 2:**
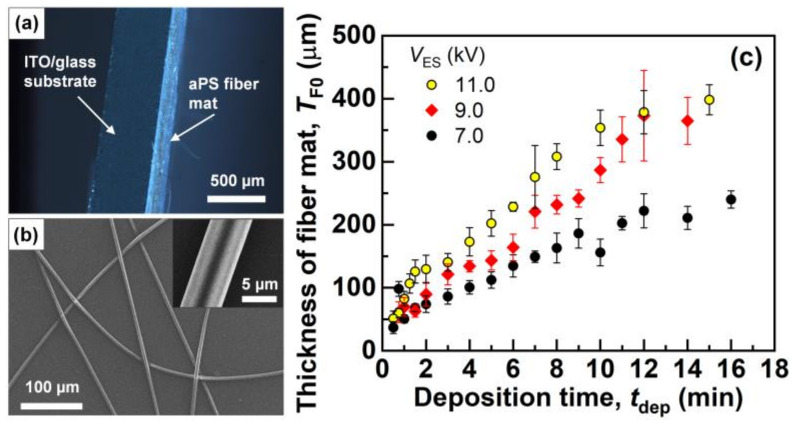
(**a**) Dark-field cross-sectional optical microscopy image of the electrospun aPS fiber mat produced with *V*_ES_ = 9.0 kV and *t*_dep_ = 9 min. (**b**) An FESEM image of the individual aPS fibers. The inset shows an enlarged FESEM image of a single fiber. (**c**) The average thickness of each fiber mat. The error bars represent standard deviations.

Figure 2a shows a cross-sectional dark-field optical microscopy image of the as-electrospun aPS fiber mat. A thin layer of the fiber mat had formed on the ITO-coated glass substrate without any significant air gaps. Figure 2b shows the FESEM image of the individual aPS fibers, which showed uniform diameters without beaded non-uniform geometries. The average diameters of the individual fibers electrospun with *V*_ES_ of 7.0, 9.0, and 11.0 kV were 5.8 ± 0.5, 6.1 ± 0.3, and 5.2 ± 0.8 μm (the error bars represent standard deviations), respectively. Figure 2c shows the average thickness of each fiber mat produced with different values of *t*_dep_ and *V*_ES_. The average thickness increased as *t*_dep_ increased at *t*_dep_ ≤ 10 min, then slowed the increase at *t*_dep_ > ~10 min. This slowed increase in thickness can be attributed to the fact that the highly charged fiber mat deposited in advance repulsed the newly deposited fibers charged with the same polarity (positive). The higher *V*_ES_ produced thicker fiber mats despite having the same *t*_dep_, which was attributed to the higher production rate of the fibers with increasing voltage [27], which is also supported by the higher feed rate with higher *V*_ES_.

### 3.2. Direct Electromechanical Characteristics

**Figure 3:**
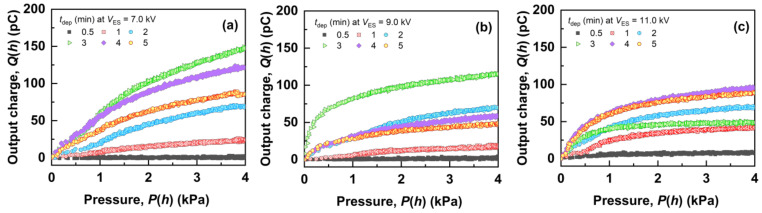
*Q*(*h*) with *P*(*h*) of the fiber mats produced with different *t*_dep_ and *V*_ES_. (**a**) 7.0, (**b**) 9.0, and (**c**) 11.0 kV.

Figure 3 shows the representative results of *Q*(*h*) with *P*(*h*) from the fiber mats produced with different *t*_dep_ and *V*_ES_. All of the results are shown in Figures S1–S3 of the Supplementary Material. The fiber mats produced with (*t*_dep_ = 0.5 min, *V*_ES_ = 7.0 kV) and (*t*_dep_ = 0.5 min, *V*_ES_ = 9.0 kV) output minimal charges, considering that they were almost uncharged (as is described in detail in Section 3.3). However, the fiber mats produced with other *t*_dep_ and *V*_ES_ output higher charges with increasing *P*(*h*). This charge output with the pressing of the fiber mats can be explained by the following mechanism: firstly, the as-electrospun aPS fiber mats were charged via electrospinning. When the charged fiber mats were pressed with the disk-shaped metallic probe, the effective surface charge density of the fiber mats increased. As a result, the inductive charge density at the probe also increased, such that the amount of change in the inductive charges before/after pressing was output. A detailed explanation of the mechanism has been described elsewhere [26], and the charging models of the mats were discussed in Section 3.3. At the lower*P*(*h*), *Q*(*h*) shows higher increase, owing to the nonlinear elastic deformation of the fiber mats [28]. The amount of *Q*(*h*) for each *V*_ES_ increased as *t*_dep_ increased to 3 min (*V*_ES_ = 7.0 kV), 3 min (*V*_ES_ = 9.0 kV), and 4 min (*V*_ES_ = 11.0 kV). After these deposition times, *Q*(*h*) decreased as *t*_dep_ increased. The reason for the peak *Q*(*h*) values is explained in Section 3.3.

The apparent piezoelectric *d* constant (*d*_app_) of each fiber mat was determined as follows [28]:

(1) $$d_{\mathrm{app}}=Q\left(h,h_{\mathrm{pre}}\right)/\left[P\left(h,h_{\mathrm{pre}}\right)\cdot S\right]$$

where,

(2) $$Q\left(h,h_{\mathrm{pre}}\right)=Q\left(h\right)-Q\left(h_{\mathrm{pre}}\right)\;\;\left(h\leq 0,h_{\mathrm{pre}}\leq 0\right)$$

(3) $$P\left(h,h_{\mathrm{pre}}\right)=P\left(h\right)-P\left(h_{\mathrm{pre}}\right)\;\;\left(h\leq 0,h_{\mathrm{pre}}\leq 0\right)$$

where $P\left(h_{\mathrm{pre}}\right)$ is the pre-pressure, $h_{\mathrm{pre}}$ is *h* when $P\left(h_{\mathrm{pre}}\right)$ is applied, and $P\left(h_{\mathrm{pre}}\right)$ was determined to be ~0.2 kPa, which was obtained from previous reports on the quasistatic direct electromechanical characterization of as-electrospun aPS fiber mats [26,29]. *d*_app_ was experimentally determined using Equations (1)–(3) and the data of *Q*(*h*) with *P*(*h*) shown in Figure 3, as well as Figures S1–S3 in the Supplementary Material.

**Figure 4:**
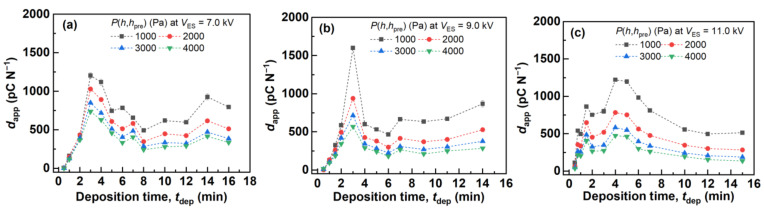
*d_a_*_pp_ measured from the aPS fiber mats produced with different *t*_dep_ and *V*_ES_. (**a**) 7.0, (**b**) 9.0, and (**c**) 11.0 kV.

Figure 4 summarizes the experimentally determined value of *d*_app_ when *P*(*h,h*_pre_) = 1, 2, 3, and 4 kPa. The *d*_app_ with different *t*_dep_ for each *V*_ES_ varied significantly and exhibited peak values at *t*_dep_ = 3 min (*V*_ES_ = 7.0 kV), *t*_dep_ = 3 min (*V*_ES_ = 9.0 kV), and *t*_dep_ = 4 min (*V*_ES_ = 11.0 kV). The results showed that changing the electrospinning conditions of *t*_dep_ and *V*_ES_ drastically changed the value of *d*_app_. The *d*_app_ of the fiber mat produced with *t*_dep_ = 3 min and *V*_ES_ = 9.0 kV was ~1600 pC N^−1^ under *P*(*h,h*_pre_) = 1 kPa and $P\left(h_{\mathrm{pre}}\right)$ ~0.2 kPa; the *d*_app_ value was higher than the previsously reported *d*_app_ of the as-electrospun aPS fiber mat, ~730 pC N^−1^ [30] and ~1020 pC N^−1^ [28], measured using the same technique and conditions.

Iumsrivun et al. [25] reported that the direct electromechanical responses from the as-electrospun aPS fiber mats originated from stored real charges (such as surface and space charges) in the fiber mats supplied via electrospinning. Ishii et al. [29] demonstrated that when the fibers were electrospun with a positive voltage, the positive and negative charges were generally stored in the upper and lower parts of the fiber mats, respectively. Additionally, when the fiber mat is assumed to be an infinitely wide rectangular structure and charged with effective surface charge densities of +*σ*_Eff0_ and −*σ*_Eff0_ at the upper and lower surfaces, respectively, *d*_app_ can be theoretically defined as [26]:

(4) $$d_{\mathrm{app}}\approx \frac{T_{F0}\sigma_{\mathrm{Eff}0}}{Y_{D}\left(h-h_{\mathrm{pre}}\right)\left(T_{F0}+h\right)}\;\;\left(h\leq 0,h_{\mathrm{pre}}\leq 0\right)$$

where,

(5) $$Y_{D}\left(h-h_{\mathrm{pre}}\right)=\frac{P\left(h,h_{\mathrm{pre}}\right)\left(T_{F0}+h_{\mathrm{pre}}\right)}{\left(h_{\mathrm{pre}}-h\right)}\;\;(h\leq 0,h_{\mathrm{pre}}\leq 0)$$

where *Y*_D_(*h*-*h*_pre_) is the secant modulus of elasticity of the fiber mat, determined under the conditions of *P*(*h,h*_pre_) and *h*_pre_-*h*. Assuming that *T*_F0_ >> *h*_pre_ and *T*_F0_ >> *h*, Equation (4) can be approximated as follows:

(6) $$d_{\mathrm{app}}\approx \frac{\sigma_{\mathrm{Eff}0}}{Y_{D}\left(h-h_{\mathrm{pre}}\right)}\;\;(h_{\mathrm{pre}}\ll T_{F0},h\ll T_{F0})$$

Equation (6) demonstrates that *Y*_D_(*h*-*h*_pre_) and *σ*_Eff0_ dominantly affect *d*_app_. Therefore, *Y*_D_(*h*-*h*_pre_) and *σ*_Eff0_ were investigated in order to determine why *d*_app_ with different *t*_dep_ values for each *V*_ES_ showed the peak values.

**Figure 5:**
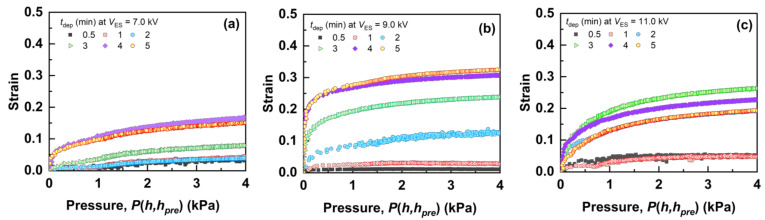
Strain–pressure characteristics of the fiber mats produced with different *t*_dep_ and *V*_ES_. (**a**) 7.0, (**b**) 9.0, and (**c**) 11.0 kV.

In order to investigate *Y*_D_(*h*-*h*_pre_), the strain–pressure characteristics of each fiber mat were measured while simultaneously conducting electromechanical measurements. Figure 5 and Figures S4–S6 in the Supplementary Material show the results of the strain–pressure characteristics. Each fiber mat showed nonlinear elastic deformation, where the degree of strain change was higher in the lower pressure range. Furthermore, the strain–pressure characteristics varied significantly for the fiber mats produced with different *t*_dep_, in contrast to those produced with the same *V*_ES_. These results indicate that the mechanical characteristics of only the fiber mats produced with different *t*_dep_ values changed significantly.

**Figure 6:**
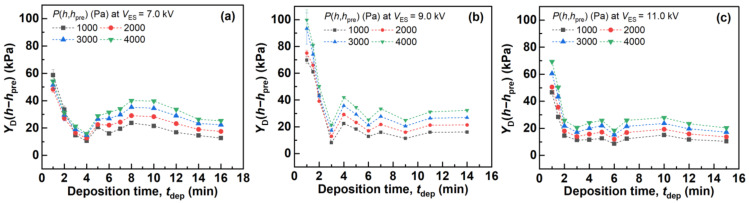
*Y*_D_(*h*-*h*_pre_) of the fiber mats produced with different *t*_dep_ and *V*_ES_. (**a**) 7.0, (**b**) 9.0, and (**c**) 11.0 kV.

As the pressure and strain are *P*(*h*,*h*_pre_) and $\left(h_{\mathrm{pre}}-h\right)/\left(T_{F0}{+h}_{\mathrm{pre}}\right)$, respectively, *Y*_D_(*h*-*h*_pre_) can be calculated from the reciprocal of the tilting between each plot and the origin of the strain–pressure characteristics shown in Figure 5, based on Equation (5). Figure 6 shows the evaluated *Y*_D_(*h*-*h*_pre_) with different *P*(*h*,*h*_pre_) values of the fiber mats produced with different *t*_dep_ and *V*_ES_. *Y*_D_(*h*-*h*_pre_) evaluated under *P*(*h*,*h*_pre_) = 1, 2, 3, and 4 kPa were found to be less than 100 kPa, which indicated that the fiber mats were soft. Furthermore, *Y*_D_(*h*-*h*_pre_) increased as *P*(*h*,*h*_pre_) increased, owing to the densification of each fiber mat due to the increase in the applied pressure. Moreover, *Y*_D_(*h*-*h*_pre_) with different *t*_dep_ values varied significantly when the fiber mats were produced with the same *V*_ES_; that is, *Y*_D_(*h*-*h*_pre_) decreased significantly as *t*_dep_ increased to *t*_dep_ = 3 min (*V*_ES_ = 7.0 kV), *t*_dep_ = 3 min (*V*_ES_ = 9.0 kV), and *t*_dep_ = 2 min (*V*_ES_ = 11.0 kV). At this stage, *Y*_D_(*h*-*h*_pre_) started to saturate. The origin of this trend is discussed in Section 3.4.

**Figure 7:**
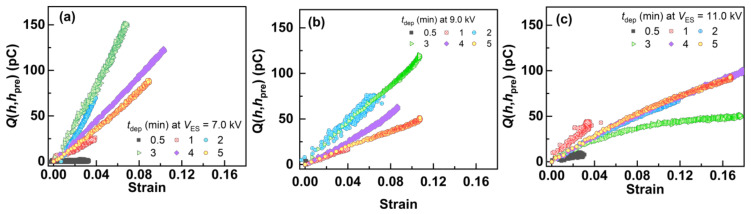
*Q*(*h*,*h*_pre_) with different strains of the fiber mats produced with different *t*_dep_ and *V*_ES_. (**a**) 7.0, (**b**) 9.0, and (**c**) 11.0 kV].

In order to investigate *σ*_Eff0_, *Q*(*h*,*h*_pre_) was plotted with different strains of each fiber mat, as shown in Figure 7. Ishii et al. [26] proposed the following equation relating to the direct electromechanical characteristics of the as-electrospun aPS fiber mats:

(7) $$Q\left(h,h_{\mathrm{pre}}\right)\approx -\frac{T_{F0}\sigma_{\mathrm{Eff}0}S}{T_{F0}+h_{\mathrm{pre}}}\cdot \frac{h}{T_{F0}-h_{\mathrm{pre}}+h}\;\;\left(h\leq 0,h_{\mathrm{pre}}\leq 0\right)$$

**Figure 8:**
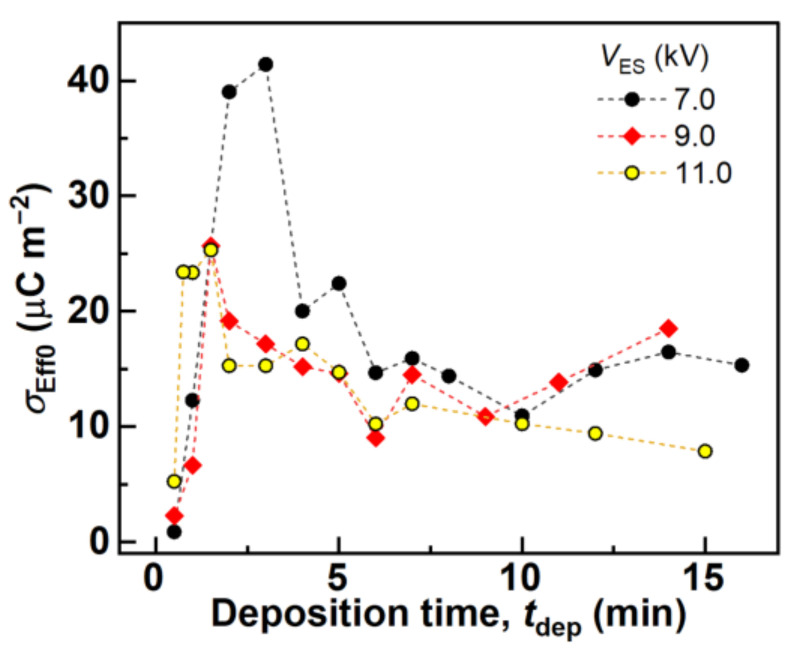
Calculated *σ*_Eff0_ of the fiber mats produced with different *t*_dep_ and *V*_ES_.

Assuming *T*_F0_ >> *h*_pre_ and *T*_F0_ >> *h*, Equation (7) can be approximated as follows:

(8) $$Q\left(h,\;h_{\mathrm{pre}}\right)\approx -\sigma_{\mathrm{Eff}0}S\frac{h}{T_{F0}}\left(h\leq 0\right)$$

where −*h*/*T*_F0_ yields the strain of the fiber mat, such that *Q*(*h*,*h*_pre_) increases linearly with the strain for *T*_F0_ >> *h*_pre_ and *T*_F0_ >> *h*. Therefore, *σ*_Eff0_ can be calculated from the tilt of the plot of *Q*(*h*,*h*_pre_) with the strain. Figure 7 and Figures S7–S9 of the Supplementary Material show the *Q*(*h*,*h*_pre_)–strain characteristics of the fiber mats produced with different *t*_dep_ and *V*_ES_, which varied significantly with different *t*_dep_ values for each *V*_ES_. In addition, the *Q*(*h*,*h*_pre_) of each fiber mat increased linearly as the strain increased at each low-strain range. *σ*_Eff0_ was evaluated by fitting the plots of each *Q*(*h*,*h*_pre_)–strain characteristic using Equation (8), as shown in Figure 8. *σ*_Eff0_ with different *t*_dep_ values for each *V*_ES_ showed peak values for 0 min < *t*_dep_ ≤ 4 min. Over the *t*_dep_ range, *σ*_Eff0_ decreased or showed an approximately saturating value, which indicated that *σ*_Eff0_ changed only by varying *t*_dep_; that is, *t*_dep_ changed the density of the real charges stored in the fiber mat. Section 3.3 describes why the evaluated *σ*_Eff0_ exhibited this trend.

**Figure 9:**
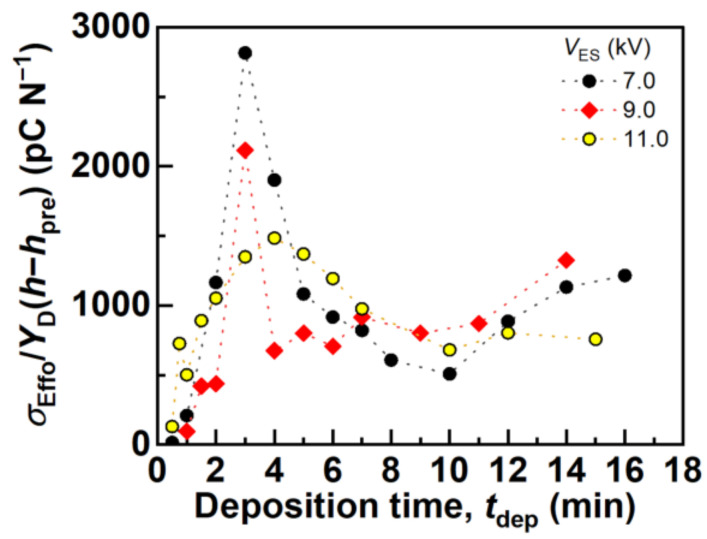
Calculated *σ*_Eff0_/*Y*_D_(*h*-*h*_pre_) of the fiber mats produced with different *t*_dep_ and *V*_ES_. Here, *Y*_D_(*h*-*h*_pre_) with *P*(*h*) = 1 kPa was used for the calculation.

Next, *σ*_Eff0_/*Y*_D_(*h*-*h*_pre_) was calculated using the evaluated *σ*_Eff0_ and *Y*_D_(*h*-*h*_pre_) with *P*(*h*,*h*_pre_) = 1 kPa in order to discuss the relationship between the measured *d*_app_ and *σ*_Eff0_/*Y*_D_(*h*-*h*_pre_) based on Equation (6), which are summarized in Figure 9. Here, *Y*_D_(*h*-*h*_pre_) determined under *P*(*h*,*h*_pre_) = 1 kPa was used for the calculation, considering that *P*(*h*,*h*_pre_) = 1 kPa was the closest condition to the assumption *T*_F0_ >> *h*_pre_ and *T*_F0_ >> *h* among *P*(*h*,*h*_pre_) = 1, 2, 3, and 4 kPa. Upon comparing *d*_app_ with *P*(*h*,*h*_pre_) = 1 kPa and *σ*_Eff0_/*Y*_D_(*h*-*h*_pre_) in Figure 4 and Figure 9, respectively, with different *t*_dep_ for each *V*_ES_, both showed similar trends; that is, both *d*_app_ and *σ*_Eff0_/*Y*_D_(*h*-*h*_pre_) with different *t*_dep_ for each *V*_ES_ showed peak values at *t*_dep_ = 3 min (*V*_ES_ = 7.0 kV), *t*_dep_ = 3 min (*V*_ES_ = 9.0 kV), and *t*_dep_ = 4 min (*V*_ES_ = 11.0 kV). This indicated that the *d*_app_ of the fiber mats produced with different *t*_dep_ for each *V*_ES_ showed a peak value, considering that the *σ*_Eff0_/*Y*_D_(*h*-*h*_pre_) value of these fiber mats exhibited peak values with different *t*_dep_ values for each *V*_ES_. Therefore, both *σ*_Eff0_ and *Y*_D_(*h*-*h*_pre_) dominantly affected the *d*_app_ values according to Equation (6) under the assumption that *T*_F0_ >> *h*_pre_ and *T*_F0_ >> *h*, and *σ*_Eff0_, *Y*_D_(*h*-*h*_pre_), and the resulting *d*_app_ varied significantly under different electrospinning conditions of *t*_dep_ and *V*_ES_. Additionally, the above-mentioned trend correspondence between the measured *d*_app_ and calculated *σ*_Eff0_/*Y*_D_(*h*-*h*_pre_) strongly supports the validity of Equation (6). The following section discusses the reason why the evaluated *Y*_D_(*h*-*h*_pre_) and *σ*_Eff0_ showed peak values with different *t*_dep_.

### 3.3. Charge Distribution in the Fiber Mat

**Figure 10:**
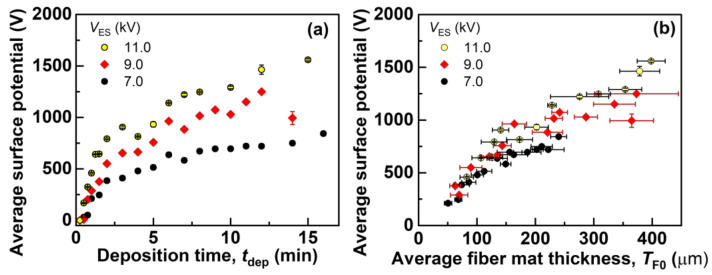
Average surface potential of each fiber mat produced with different *t*_dep_ and *V*_ES_ as a function of (**a**) *t*_dep_ and (**b**) the average fiber mat thickness. The error bars represent standard deviations.

In order to understand why *σ*_Eff0_ and *Y*_D_(*h*-*h*_pre_) show peak values with different *t*_dep_ for each *V*_ES_, it is necessary to understand the distribution of the stored real charges and geometrical characteristics of the fiber mats. Figure 10a shows the average surface potential of each fiber mat, which can be categorized into three phases: in Phase I, the surface potential is nearly zero; in Phase II, the surface potential significantly increases as *t*_dep_ increases; in Phase III, the surface potential slows its increase. Table 2 summarizes the values of *t*_dep_ corresponding to the three phases. Figure 10b shows the surface potential with an average thickness of each fiber mat, where the turning points from Phase II to Phase III in Figure 10a [*t*_dep_ = 2 min (*V*_ES_ = 7.0, 9.0, and 11.0 kV)] correspond to *T*_F0_ = 74 μm (*V*_ES_ = 7.0 kV), 90 μm (*V*_ES_ = 9.0 kV), and 110 μm (*V*_ES_ = 11.0 kV). Similarly to the trend of the surface potential with *t*_dep_, the surface potential increased significantly with *T*_F0_ in Phase II, and the surface potential slowed its increase in Phase III, thereby indicating that the distribution of the stored real charges is different and can be categorized into three models.

**Figure 11:**
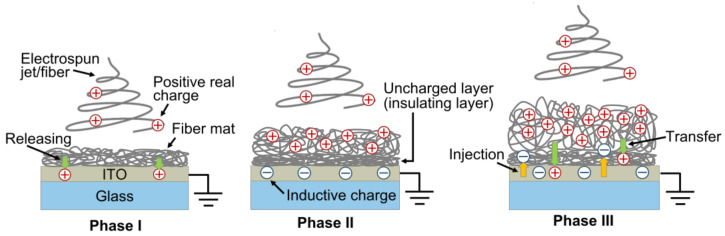
Charging models of the as-electrospun aPS fiber mat in Phases I, II, and III.

Figure 11 shows the charging models of the as-electrospun aPS fiber mat at Phases I, II, and III. In this study, the positive high voltages were used for electrospinning, such that the electrospun jets of the solution and the resulting fibers after the evaporation of the solvent were charged positively, owing to the injection of positive real charges. In Phase I (Figure 11), the positively charged fibers were deposited on the ITO bottom electrode in direct contact, such that most of the positive real charges were released to the ITO electrode. As a result, the produced fiber mat became uncharged, leading to approximately zero surface potential. Considering that the uncharged fiber layer acted as an uncharged insulating layer, once the uncharged fibers covered the entire surface of the ITO electrode and reached a specific thickness, the newly supplied positively charged fibers were deposited on the insulating fiber layer without releasing the real charges stored in the fibers owing to the presence of the insulating fiber layer (Phase II, Figure 11). As a result, the surface potential of the resulting fiber mat increased significantly. In Phase II, negative inductive charges were induced in the ITO bottom electrode owing to the stacked positively charged fibers. In Phase III (Figure 11), electrical breakdown occurred because of (i) excessively stacked positive charges stored in the fibers, and (ii) the reduced distance between the stored positive charges and induced negative charges across the insulating fiber layer owing to the increased electrostatic attraction between these charges. Here, the *t*_dep_ value that induced the electrical breakdown should be determined using Paschen’s law. The electrical breakdown can cause the following two phenomena: (i) the induced negative charges in the ITO electrode are injected into the insulating fiber layer or/and positively charged fiber layers, which decreases the total quantity of electric charges stored in the fiber mat; and (ii) the stored positive real charges in the positively charged fiber layer are transferred to the insulating fiber mat layer and/or ITO electrode, which decreases the total quantity of electric charges and the distance between the positive charges and the ITO electrode grounded electrically during the surface potential measurement. These phenomena in Phase III can slow the increase in the surface potential with *t*_dep_.

**Figure 12:**
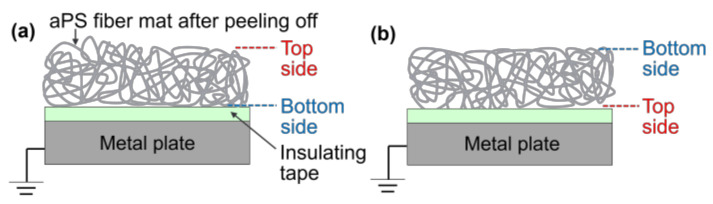
Schematic of the surface potential measurement for the fiber mats after peeling them off and placing them in the (**a**) forward and (**b**) opposite arrangements.

In order to further verify the results of the proposed charging models in Phases II and III, the surface potential of the fiber mats produced with different *t*_dep_ was measured after peeling them from each ITO/glass substrate and placing them on an insulating-tape-covering metal plate with forward and opposite arrangements (Figure 12). Then, the surface potential was measured while the metal plate was electrically grounded. The metal plate was covered with insulating tape in order to prevent the stored real charges in each fiber mat from releasing into the plate. The surface potential of the insulating tape without the fiber mat was confirmed to be zero before each measurement.

Table 3 summarizes the average surface potentials measured from four samples produced under the same conditions, alongside the average and standard deviation. For all of the individual fiber mats produced with different *t*_dep_, the absolute values of the surface potentials measured with the forward arrangement demonstrated higher values compared to those with the opposite arrangement. This result supports the fact that positive real charges are generally held in the upper part of the fiber mats. The surface potential measured with the opposite arrangement demonstrated positive and negative values of 0.75 min ≤ *t*_dep_ ≤ 2 min (the *t*_dep_ range corresponding to Phase II) and 3 min ≤ *t*_dep_ ≤ 15 min (the *t*_dep_ range corresponding to Phase III). This result shows that the lower part of the fiber mats at 0.75 min ≤ *t*_dep_ ≤ 2 min holds few negative real charges, corresponding to the charging model of Phase II (Figure 11); and the lower part of the ﬁber mats at 3 min ≤ *t*_dep_ ≤ 15 min holds larger numbers of negative real charges than those at 0.75 min ≤ *t*_dep_ ≤ 2 min, corresponding to the charging model of Phase III (Figure 11). These results strongly support the proposed charge models for Phases II and III. Table 3 also summarizes the amount of charge measured from the fiber mats produced with different *t*_dep_ after peeling them from each ITO/glass substrate. Each value of the amount of charge showed a positive value, such that the number of positive real charges was larger than that of the negative real charges in each fiber mat in Phases II and III. This result is also supported by the fact that the absolute values of the surface potentials measured with the forward arrangement demonstrated higher values compared to those with the opposite arrangement for 3 min ≤ *t*_dep_ ≤ 15 min.

### 3.4. Structure Inside the Fiber Mat

**Figure 13:**
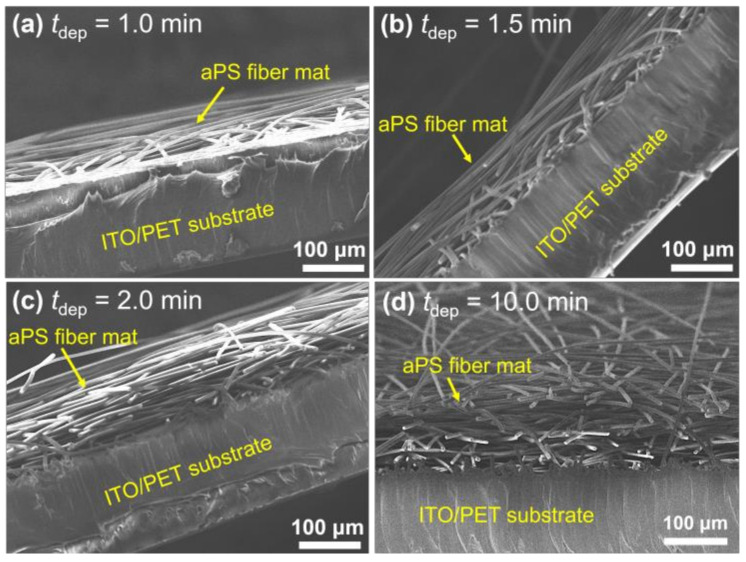
Cross-sectional FESEM images of the as-electrospun aPS fiber mats produced with *V*_ES_ = 11.0 kV and different *t*_dep_: (**a**) 1.0, (**b**) 1.5, (**c**) 2.0 and (**d**) 10.0 min.

In order to investigate why the measured *Y*_D_(*h*-*h*_pre_) decreased as *t*_dep_ increased before saturating at, for example, 0 min < *t*_dep_ ≤ 2 min (*V*_ES_ = 11.0 kV), as shown in Figure 6, the cross-sectional FESEM images of the electrospun aPS fiber mats produced with different *t*_dep_, as shown in Figure 13, were analyzed. In the fiber mats produced with *t*_dep_ = 1.0 and 1.5 min, of which the *Y*_D_(*h*-*h*_pre_) was significantly higher than that when *t*_dep_ ≥ 2.0 min, the fibers generally lay parallel to the ITO/PET substrate and formed a thin, several-layered fiber mat. Additionally, the fibers at the bottom of the fiber mats stuck and lay parallel to the ITO electrode. Conversely, in the fiber mats produced when *t*_dep_ ≥ 2.0 min, of which the*Y*_D_(*h*-*h*_pre_) was significantly lower than those produced when *t*_dep_ = 1.0 and 2.0 min, the fibers formed a thick multi-layered fiber mat with the individual fibers lying parallel to the ITO/PET substrate. In thin multi-layered fiber mats, when *t*_dep_ = 1.0 and 1.5 min, the stiffness of the individual fibers strongly affected the mechanical properties of the fiber mats, such that *Y*_D_(*h*-*h*_pre_) exhibited higher values. In thick, multi-layered fiber mats, when *t*_dep_ ≥ 2.0 min, the sparse stacking structure significantly affected the mechanical properties of the fiber mats compared to the stiffness of the individual fibers, such that *Y*_D_(*h*-*h*_pre_) exhibited lower values.

## 4. Conclusions

In this study, the direct electromechanical properties of the as-electrospun aPS fiber mats produced with different *t*_dep_ and *V*_ES_ values were investigated. The resulting *d*_app_ of the fiber mats demonstrated a specific peak value for *t*_dep_ as high as ~1600 pC N^−1^ under 1-kPa pressure application after the application of ~0.2-kPa pre-pressure, although the *d*_app_ of the fiber mats produced with some conditions was nearly zero pC N^−1^. The origin of the specific peak value was investigated by evaluating *Y*_D_(*h*-*h*_pre_) and *σ*_Eff0_ for each fiber mat. The results showed that *Y*_D_(*h*-*h*_pre_) and *σ*_Eff0_ exhibited significantly different values as *t*_dep_ varied for each *V*_ES_, whereas *σ*_Eff0_/*Y*_D_(*h*-*h*_pre_) determined the peak position of *d*_app_ with *t*_dep_. Furthermore, models of the charge distribution in the fiber mat with different *t*_dep_ were established by measuring the surface potential of each fiber mat and the geometrical arrangements, then classifying the models into three phases. As a result, the three phases clearly explained the characteristics of the significant changes in *Y*_D_(*h*-*h*_pre_) and *σ*_Eff0_ as *t*_dep_ varied, along with the structure of the fiber mats. The findings of this study provide guidance for the production of fiber mats exhibiting significantly improved direct electromechanical properties; hence, they can pave the way for high-performance pressure sensors that are flexible, lightweight, and can be produced on a large scale at a low cost.

## Figures and Tables

**Table 1:** Electrospinning conditions.

Applied Voltage [kV]	Feed Rate [mL/h]	Deposition Time [Min]	Temperature [°C]	Humidity [%RH]
7.0	0.65	0.25, 0.5, 0.75, 1, 2, 3, 4, 5, 6, 7, 8, 10, 12, 14, 16	28–29	25–26
9.0	1.40	0.25, 0.5, 0.75, 1, 1.5, 2, 3, 4, 5, 6, 7, 9, 11, 14	26–28	27–30
11.0	2.60	0.25, 0.5, 0.75, 1, 1.5, 2, 3, 4, 5, 6, 7, 10, 12, 15	23–24	24–26

**Table 2:** Ranges of *t*_dep_ corresponding to Phases I, II, and III.

*V*_ES_ [kV]	Phase I	Phase II	Phase III
7.0	0 min < *t*_dep_ ≤ 0.75 min	0.75 min < *t*_dep_ ≤ 2 min	2 min < *t*_dep_
9.0	0 min < *t*_dep_ ≤ 0.5 min	0.5 min < *t*_dep_ ≤ 2 min	2 min < *t*_dep_
11.0	0 min < *t*_dep_ < 0.5 min	0.5 min ≤ *t*_dep_ ≤ 2 min	2 min < *t*_dep_

**Table 3:** Surface potentials of the fiber mats produced with different *t*_dep_ in *V*_ES_ = 11.0 kV after peeling them from each ITO/glass substrate and placing them in the forward and opposite arrangements. Here, the errors represent the standard deviations from four samples produced under the same conditions.

*t*_dep_ [min]	Surface Potential after Peeling off [V]		Amount of Charge [nC]
	Forward	Opposite	
0.75	218 ± 84	118 ± 52	6.4 ± 2.0
1	182 ± 44	110 ± 38	5.4 ± 0.9
1.5	256 ± 27	125 ± 22	8.3 ± 2.9
2	464 ± 52	401 ± 83	15.4 ± 1.2
3	599 ± 105	−483 ± 46	22.6 ± 1.2
4	688 ± 67	−362 ± 33	22.4 ± 1.0
5	684 ± 26	−220 ± 29	19.1 ± 1.0
6	635 ± 92	−288 ± 48	23.6 ± 2.9
7	255 ± 32	−214 ± 42	10.7 ± 1.0
8	218 ± 42	−202 ± 83	13.4 ± 2.0
10	366 ± 82	−397 ± 85	11.2 ± 0.5
12	305 ± 72	−287 ± 63	13.1 ± 2.0
15	503 ± 44	−423 ± 55	9.9 ± 2.0

## Data Availability

The data that support the findings of this study are available within the article and its Supplementary Material.

## Supplementary Materials

The following supporting information can be downloaded at: https://www.mdpi.com/article/10.3390/polym14091840/s1, Figure S1: *Q*(*h*) with *P*(*h*) from the fiber mats produced with *V*_ES_=7.0 kV and different *t*_dep_. (a) *t*_dep_ = 0.5, 1, 2, 3 min, (b) *t*_dep_ = 4, 5, 6, 7 min, and (c) *t*_dep_ = 8, 10, 12, 14, 16 min.; Figure S2: *Q*(*h*) with *P*(*h*) from the fiber mats produced with *V*_ES_=9.0 kV and different *t*_dep_. (a) *t*_dep_ = 0.5, 1, 1.5, 2, 3 min and (b) *t*_dep_ = 4, 5, 6, 7, 9, 11, 14 min.; Figure S3: *Q*(*h*) with *P*(*h*) from the fiber mats produced with *V*_ES_=11.0 kV and different *t*_dep_. (a) *t*_dep_ = 0.5, 0.75, 1, 1.5, 2, 3, and 4 min and (b) *t*_dep_ = 5, 6, 7, 10, 12, and 15 min.; Figure S4: Strain–pressure characteristics of the fiber mats produced with *V*_ES_=7.0 kV and different *t*_dep_. (a) *t*_dep_ = 0.5, 1, 2, and 3 min, (b) *t*_dep_ = 4, 5, 6, and 7 min, and (c) *t*_dep_ = 8, 10, 12, 14, and 16 min.; Figure S5: Strain–pressure characteristics of the fiber mats produced with *V*_ES_=9.0 kV and different *t*_dep_. (a) *t*_dep_ = 0.5, 1, 1.5, 2, and 3 min and (b) *t*_dep_ = 4, 5, 6, 7, 9, 11, and 14 min.; Figure S6: Strain–pressure characteristics of the fiber mats produced with *V*_ES_=11.0 kV and different *t*_dep_; (a) *t*_dep_ = 0.5, 0.75, 1, 1.5, 2, 3, and 4 min and (b) *t*_dep_ = 5, 6, 7, 10, 12, and 15 min.; Figure S7: Strain–pressure characteristics of the fiber mats produced with *V*_ES_=7.0 kV and different *t*_dep_; (a) *t*_dep_ = 0.5, 1, 2, and 3 min, (b) *t*_dep_ = 4, 5, 6, and 7 min, and (c) *t*_dep_ = 8, 10, 12, 14, and 16 min.; Figure S8: Strain–pressure characteristics of the fiber mats produced with *V*_ES_=9.0 kV and different *t*_dep_. (a) *t*_dep_ = 0.5, 1, 1.5, 2, and 3 min and (b) *t*_dep_ = 4, 5, 6, 7, 9, 11, and 14 min.; Figure S9: Strain–pressure characteristics of the fiber mats produced with *V*_ES_=11.0 kV and different *t*_dep_. (a) *t*_dep_ = 0.5, 0.75, 1, 1.5, 2, 3, and 4 min and (b) *t*_dep_ = 5, 6, 7, 10, 12, and 15 min.
