# Supplementary material for: Variable Direct Electromechanical Properties of As-Electrospun Polystyrene Microfiber Mats with Different Electrospinning Conditions
Source: Polymers (Basel). 2022 Apr 29;14(9):1840. doi: 10.3390/polym14091840 (PMC9105862; doi:10.3390/polym14091840)
Supplement: Supplementary file 1 [file polymers-14-01840-s001.zip › polymers-1657722-supplementary.pdf]

polymers

MDPI

Supplementary Material

Variable Direct Electromechanical Properties of As-Electrospun Polystyrene Microfiber Mats with Different Electrospinning Conditions

Chonthicha Iumsrivun, Kazuki Matsuda, Shunsaku Ohkubo and Yuya Ishii *

Faculty of Fiber Science and Engineering, Kyoto Institute of Technology, Kyoto 606-8585, Japan; d9851501@edu.kit.ac.jp (C.I.); m0651020@edu.kit.ac.jp (K.M.); m0651004@edu.kit.ac.jp (S.O.)
* Correspondence: yishii@kit.ac.jp

S-1. $Q(h)$ with $P(h)$ of each fiber mat

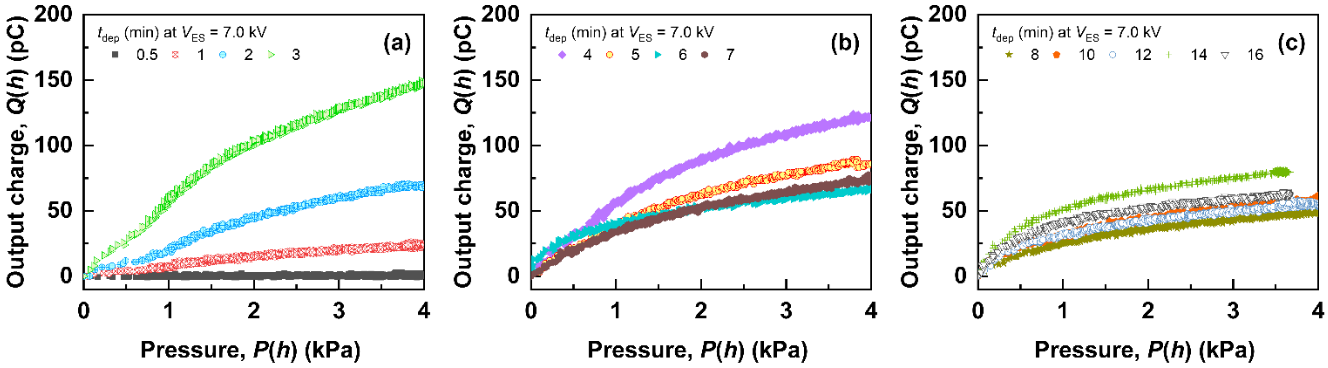

Figure S1. $Q(h)$ with $P(h)$ from the fiber mats produced with V_{ES} = 7.0 kV and different t_{dep}. (**a**) t_{dep} = 0.5, 1, 2, 3 min, (**b**) t_{dep} = 4, 5, 6, 7 min, and (**c**) t_{dep} = 8, 10, 12, 14, 16 min.

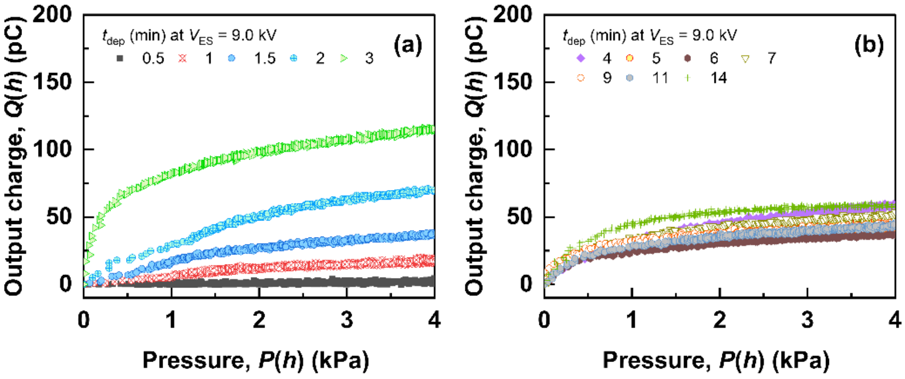

Figure S2. $Q(h)$ with $P(h)$ from the fiber mats produced with V_{ES} = 9.0 kV and different t_{dep}. (**a**) t_{dep} = 0.5, 1, 1.5, 2, 3 min and (**b**) t_{dep} = 4, 5, 6, 7, 9, 11, 14 min.

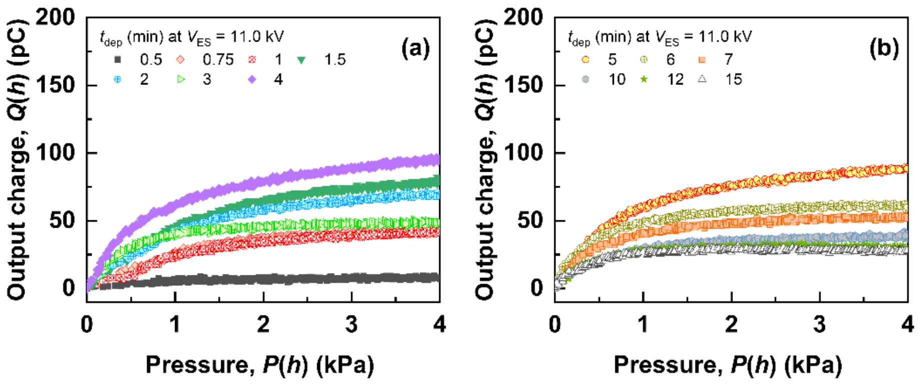

Figure S3. $Q(h)$ with $P(h)$ from the fiber mats produced with V_{ES} = 11.0 kV and different t_{dep}. (**a**) t_{dep} = 0.5, 0.75, 1, 1.5, 2, 3, and 4 min and (**b**) t_{dep} = 5, 6, 7, 10, 12, and 15 min.

S-2. Strain–pressure characteristics of each fiber mat

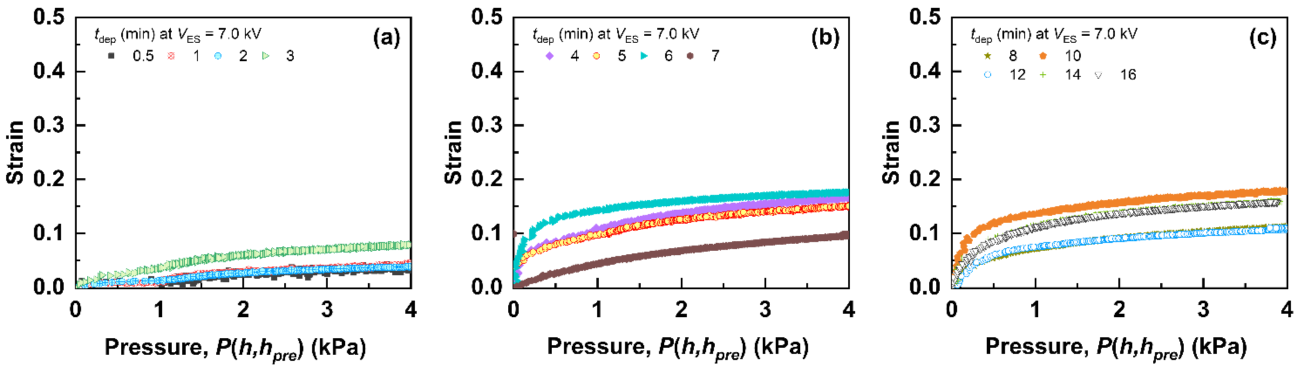

Figure S4. Strain–pressure characteristics of the fiber mats produced with V_{ES} = 7.0 kV and different t_{dep}. (**a**) t_{dep} = 0.5, 1, 2, and 3 min, (**b**) t_{dep} = 4, 5, 6, and 7 min, and (**c**) t_{dep} = 8, 10, 12, 14, and 16 min.

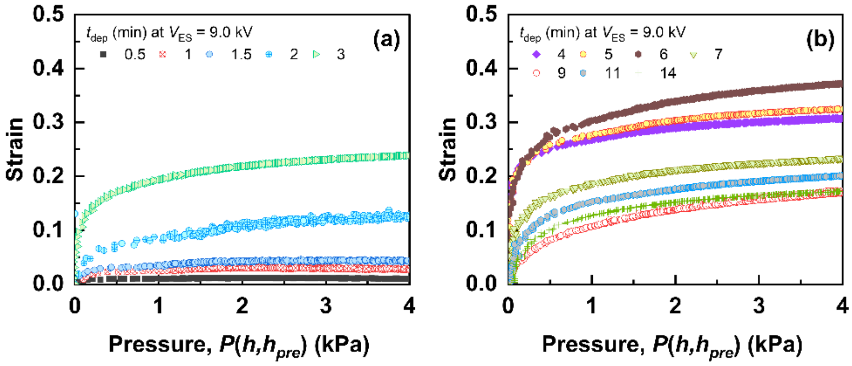

Figure S5. Strain–pressure characteristics of the fiber mats produced with V_{ES} = 9.0 kV and different t_{dep}. (**a**) t_{dep} = 0.5, 1, 1.5, 2, and 3 min and (**b**) t_{dep} = 4, 5, 6, 7, 9, 11, and 14 min.

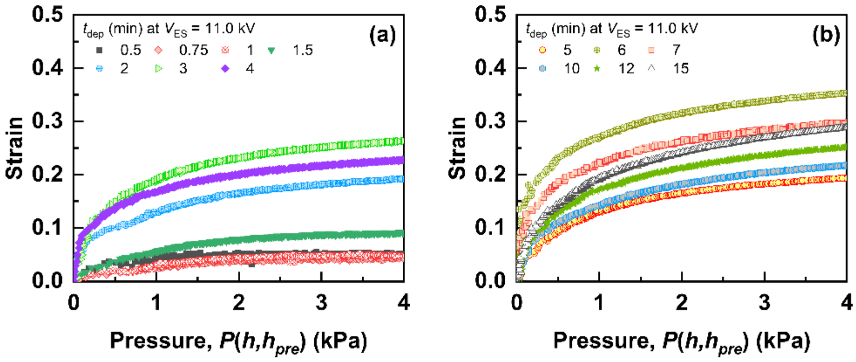

Figure S6. Strain–pressure characteristics of the fiber mats produced with V_{ES} = 11.0 kV and different t_{dep}; (**a**) t_{dep} = 0.5, 0.75, 1, 1.5, 2, 3, and 4 min and (**b**) t_{dep} = 5, 6, 7, 10, 12, and 15 min.

S-3. $Q(h,h_{pre})$ with different strains of each fiber mats

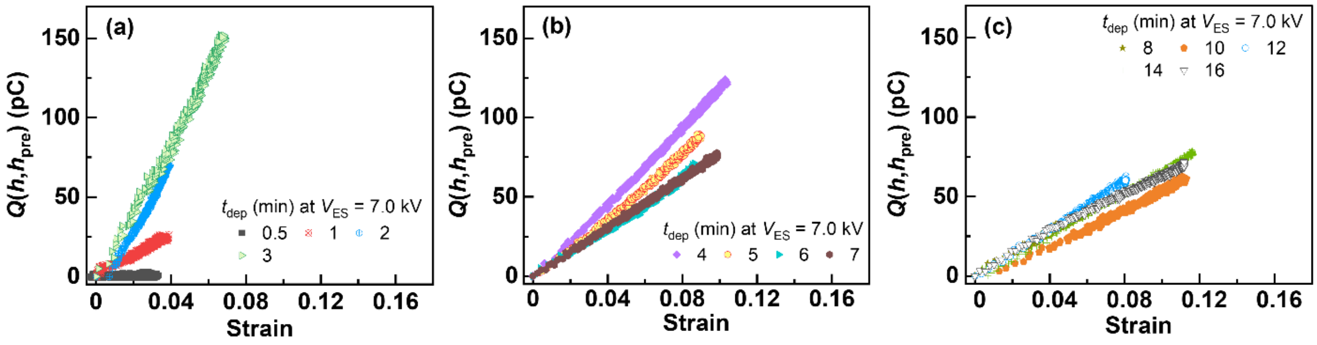

Figure S7. Strain–pressure characteristics of the fiber mats produced with V_{ES} = 7.0 kV and different t_{dep}; (**a**) t_{dep} = 0.5, 1, 2, and 3 min, (**b**) t_{dep} = 4, 5, 6, and 7 min, and (**c**) t_{dep} = 8, 10, 12, 14, and 16 min.

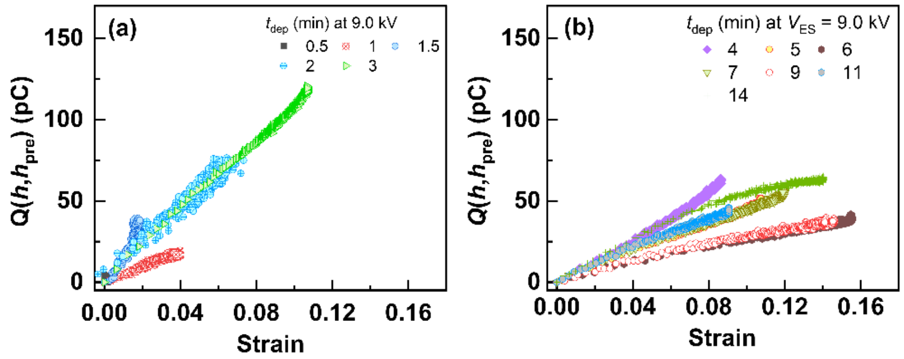

Figure S8. Strain–pressure characteristics of the fiber mats produced with V_{ES} = 9.0 kV and different t_{dep}. (**a**) t_{dep} = 0.5, 1, 1.5, 2, and 3 min and (**b**) t_{dep} = 4, 5, 6, 7, 9, 11, and 14 min.

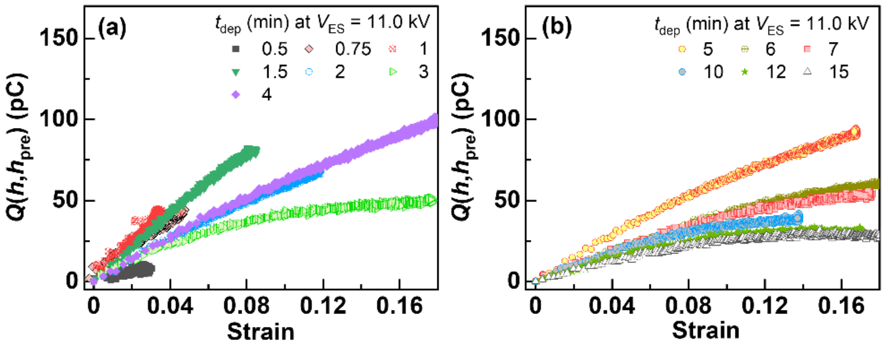

Figure S9. Strain–pressure characteristics of the fiber mats produced with V_{ES} = 11.0 kV and different t_{dep}. (**a**) t_{dep} = 0.5, 0.75, 1, 1.5, 2, 3, and 4 min and (**b**) t_{dep} = 5, 6, 7, 10, 12, and 15 min.
